# Clinicopathological spectrum of Diffuse Large B Cell lymphoma: a study targeting population yet unexplored in Pakistan

**DOI:** 10.1186/s13104-021-05768-5

**Published:** 2021-09-10

**Authors:** H. Mahmood, M. Habib, W. Aslam, S. Khursheed, S. Fatima, S. Aziz, M. Habib, M. Faheem

**Affiliations:** 1 Clinical Oncology, Nuclear Medicine Oncology & Radiotherapy Institute, Islamabad, Pakistan; 2 Hematology (Pathology), Shifa College of Medicine (Shifa Tameer-e-Millat University), Islamabad, Pakistan; 3 Hematology (Pathology), Nuclear Medicine Oncology & Radiotherapy Institute, Islamabad, Pakistan; 4 Histopathology (Pathology), Nuclear Medicine Oncology & Radiotherapy Institute, Islamabad, Pakistan; 5 Nuclear Medicine, Nuclear Medicine Oncology & Radiotherapy Institute, Islamabad, Pakistan; 6 Pathology, Nuclear Medicine Oncology & Radiotherapy Institute, Islamabad, Pakistan; 7 Restorative Dentistry, University of Malaya, Kuala Lumpur, Malaysia

**Keywords:** Diffuse Large B cell, Lymphomas, NHL, Nodal, Extra nodal

## Abstract

**Objective:**

Diffuse Large B Cell Lymphoma (DLBCL) is the most common type of Non-Hodgkin Lymphoma (NHL). The aim of this study was to assess the clinico pathological characteristics of DLBCL specifically, among the affected individuals residing in Northern areas of Pakistan who had not been previously included in major lymphoma studies due to their remote location.

**Results:**

Mean age of the patients was 49.7 years. Male: female ratio was 1.5:1. Primary site was lymph node in 99 (71.74%) patients, out of which, 36 (26.09%) patients had B symptoms and 19 (13.77%) patients had stage IV disease. 39 (28.26%) patients had primary extra nodal involvement, 4 (2.90%) patients had B symptoms and 3 (2.17%) had stage IV disease. Extra nodal sites involved in primary extra nodal DLBCL were gastrointestinal tract (GIT) 19 (48.72%), tonsils 6 (15.38%), spine 4 (10.26%), soft tissue swelling 3 (7.69%), parotid gland 2 (5.13%), thyroid 2 (5.13%) central nervous system (CNS) 1 (2.56), breast 1 (2.56%) and bone marrow 1 (2.56%). Our study revealed increased percentage of patients with nodal DLBCL in stage IV and with B symptoms. Few patients with primary extra nodal DLBCL had B symptoms and stage IV disease at presentation. GIT was the most common site of involvement in primary extra nodal DLBCL.

**Supplementary Information:**

The online version contains supplementary material available at 10.1186/s13104-021-05768-5.

## Introduction

Lymphomas are a diverse group of neoplastic disorders arising primarily in lymph nodes. They have been majorly classified into Hodgkin and Non-Hodgkin lymphomas. Hodgkin lymphomas are further subdivided into five types while NHL can be of B, T and Null cell categories having further subtypes based on their histological characteristics [1, 2]. Lymphomas can be nodal and extra nodal. Lymphoma primarily arising in lymph nodes, waldeyer’s ring, spleen and thymus are considered nodal. About 3% of lymphomas are extra nodal developing in tissues other than lymph nodes (bone, skin, thyroid, gastrointestinal tract, lung). They may even arise from sites that are devoid of lymphoid tissue. These are termed as primary extra nodal lymphomas involvement of extra nodal tissue in the presence of extensive nodal involvement suggests secondary extra nodal involvement. 80% of NHL are of B cell type. DLBCL is the commonest type of nodal and extra nodal NHL accounting for 30–40% of B cell NHL. Many studies conducted in the past have shown that the overall incidence of lymphomas has increased in developing countries, including Pakistan. The incidence of primary extra nodal lymphoma has increased almost twice as that of nodal lymphomas during the past two decades [3–8]. There are two regional cancer registries but no National Cancer Registry in Pakistan. One of the regional cancer registries is in Lahore city in province of Punjab while the other is located in Karachi city in the province of Sindh [9]. There is paucity of data on trends of nodal and extra nodal involvement in DLBCL in Northern areas of Pakistan as patients of these regions are under- represented in above mentioned regional cancer registries due to their distant location, difficulties of travelling, extremes of weather, poverty, and level of illiteracy. Health resources in far flung Northern areas are scarce as compared to cities in other provinces of Pakistan [10]. The main aim of this study is to determine the frequency and clinicopathological characteristics of nodal and extra nodal involvement in DLBCL including its most frequent site and extent of bone marrow involvement at the time of presentation.

## Main text

### Materials and methods

This descriptive study is a single center experience conducted at Nuclear Medicine Oncology and Radiotherapy Institute Islamabad (NORI) from June 2015 to December 2020. NORI hospital is one of the biggest Government cancer and research hospitals located in Islamabad, receiving samples from all over Pakistan including Northern and formal tribal areas of Pakistan which include Gilgit, Sakardu, Azad Kashmir, Abbottabad and Waziristan. Due to its geographical location, it is close to these areas. Approximately 70% of the total patients attending this hospital are residents of the above-mentioned areas while roughly 30% of patients belong to other areas of Pakistan. It is a government hospital equipped with necessary diagnostic and therapeutic facilities offering subsidized treatment modalities for low-income cancer patients neglected otherwise due to financial constraints and lack of resources. Most of patients treated at this hospital are adolescents and adults. During the study period, 260 adult lymphoma patients > 18 years of age, with majority belonging to low socioeconomic status were diagnosed and treated at this hospital. Out of 260 lymphoma patients 138 patients were diagnosed with DLBCL, making it the most common type of NHL. Patients with DLBCL as secondary disease, HIV positivity, with incomplete clinical information and immunohistochemistry data were excluded from the study. Detailed history was taken, and physical examination was conducted. CT chest, abdomen, pelvis, and bone marrow biopsies were performed and examined. Presence or absence of B symptoms (drenching night sweats, > 10% weight loss during 6 months prior to disease, fever > 38 °C) were noted. All the biopsies were examined by histopathologists. Immunohistochemistry panel of LCA, CD3, CD10, CD 20, BCL6, BCL2, MUM1, CD7, PAX5 and Ki 67 was applied on all cases and final diagnosis was made. All the cases were classified according to WHO classification of Tumors of Hematopoietic and Lymphoid tissue. Patients were completely staged according to Ann Arbor Staging System. Mean and standard deviation were calculated for quantitative variables. Percentage of nodal DLBC (involving waldeyer’s ring, spleen and thymus) and primary extra nodal DLBCL originating in extra nodal sites including GIT, (CNS), Breast, Tonsil, Thyroid, Spine, Soft tissue and Parotid gland was obtained, noted and analyzed by using SPSS 23 for Windows.

### Results

138 patients with DLBCL were included in the study. Mean age of patients was 49.7 years. There were 83 males and 55 females with a sex ratio of 1.5. Age range of patients in both male and female groups along with gender distribution is shown in Additional file 1: Figure S1. The distribution of nodal and extra nodal DLBCL in different age groups was also assessed as shown in Additional file 1: Figure S2. Chi square test was used to find correlation between age of patient and nodal and extra nodal DLBCL. No significant correlation was found between the two as shown in Additional file 1: Table S1. Please note that Figure S1, S2 and Table S1 have been included in the Additional file 1.

**Fig. 1:**
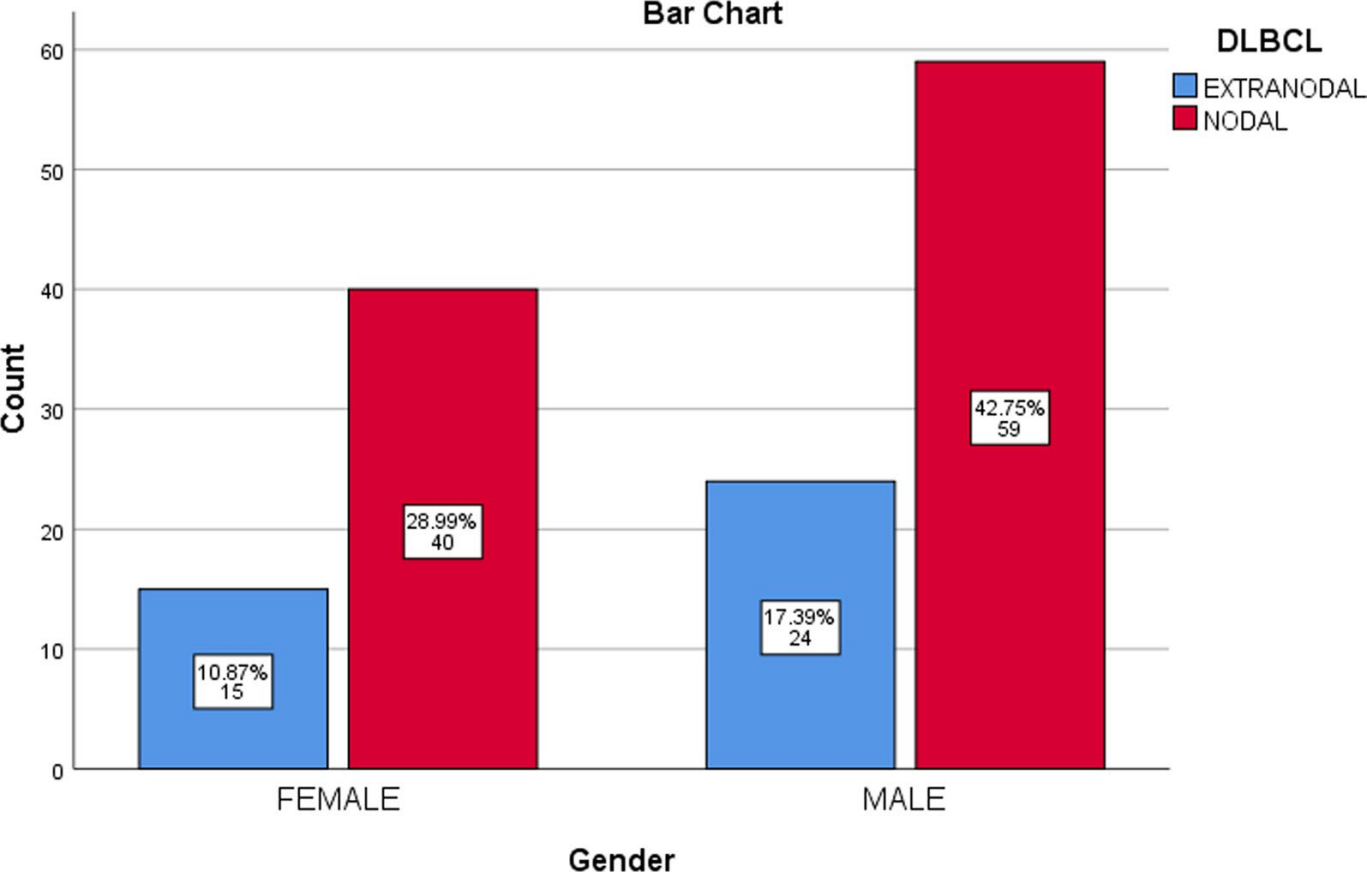
Percentage of Nodal and Extra nodal DLBCL in males and females

99 (71.74%) patients had nodal DLBCL while 39 (27.96%) had primary extra nodal disease at the time of presentation. Percentages of DLBCL in both male and female groups is shown in following Fig. 1. Positive correlation was found between gender and nodal and extra nodal involvement of DLBCL as shown in Additional file 1: Table S2 which is included in Additional file 1. Approximately equal distribution of male and female gender was seen in DLBCL involvement.

**Fig. 2:**
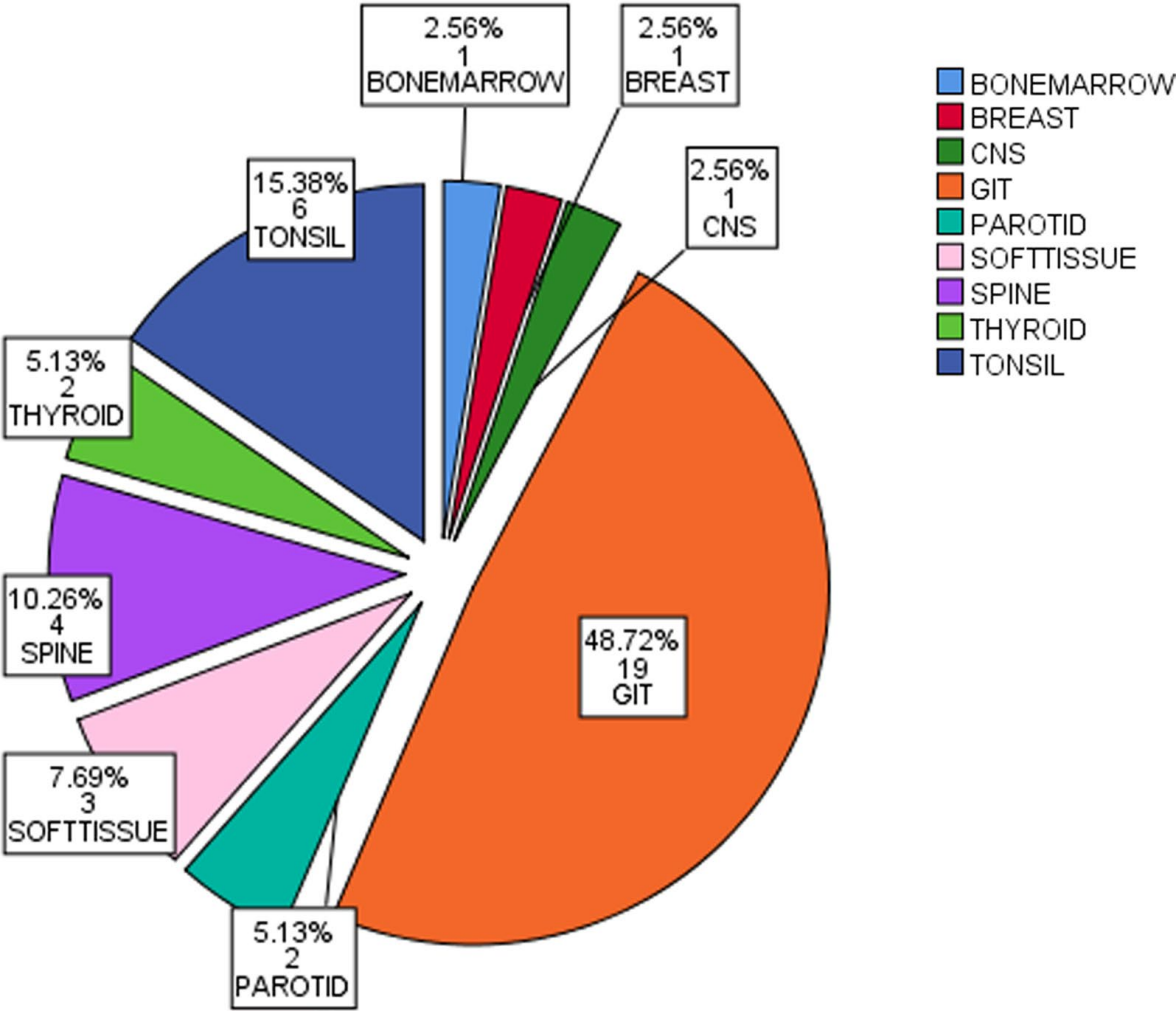
Percentage and Frequency of extra nodal sites involved in DLBCL

Gastrointestinal tract (GIT) 19 (48.72%) was the commonest site of involvement in terms of frequency followed by tonsils 6 (15.38%) as shown in Fig. 2.

Presence of B symptoms and stage 4 disease at the time of presentation was high in nodal as compared to extra nodal DLBCL Out of 99 patients of nodal DLBCL, 36 patients (26.09%) had B symptoms and 19 (13.77%) had stage 4 disease. Out of 39 patients of extra nodal DLBCL, only 4(2.90%) patients had B symptoms and 3(2.17%) extra nodal DLBCL patients had stage 4 disease at presentation as shown in Additional file 1: Figure S3 and S4 respectively, which have been included in the Additional file 1. No significant correlation was found between presence of B symptoms and stage 4 disease presentation between nodal and extra nodal DLBCL as shown in Table S3 and S4 respectively, which have been included in the Additional file 1.

## Discussion

Lymphomas are a heterogenous group of disorders with wide variation in there morphologic, geographic, and clinical spectrums. Lymphoma rank 5th to 9th among all cancers throughout the world [11]. The prevalence of lymphomas has increased in past years. NHL incidence has particularly increased and now it ranks 7th among all cancers worldwide. NHL comprises of both nodal and extra nodal lymphomas which can be primary or secondary. Incidence of extra nodal lymphomas has increased significantly especially in Pakistan [7, 12]. Different extra nodal sites are involved out of which, GIT is the most common primary extra nodal site and DLBCL is the most common type of lymphoma in primary gastrointestinal lymphoma [13]. In our study we enrolled 138 patients with DLBCL and observed the frequency of nodal and primary extra nodal DLBCL in our setup, comprising of patients from all over Pakistan with a large proportion of patients from Northern and tribal areas. In our study we found 99 patients had nodal disease out of which 36 had B-symptoms and 19 had stage 4 disease at presentation while 39 had primary extra nodal disease out of which 4 had B symptoms and 3 had stage 4 disease at presentation. GIT was the most common site of involvement (48.72%). Table 1 compares the clinicopathological characteristics of patients with DLBCL seen in our series with other studies.

**Table 1:** Comparison of spectrum of DLBCL in our series with previous studies

Parameter	Present study	A Pai [15]	X Yin [16]	A Smith [17]	A Gogia [20]	TS Waravita [27]	S Boussies [14]	D Chihara [18]
Total NHL cases	260	114	ND	4961	390	192	ND	58,230
DLBCL cases	138	43	15,882	2373	249	87	ND	21,411
Nodal Disease	99	24	ND	ND	ND	187	Over 100	9161
Extra nodal disease	39	19	15,882	ND	93	40	4	ND
Most common extra nodal site	GIT	GIT	GIT	ND	GIT	Skin	ND	Stomach
Sex ratio (Male: Female)	1.5:1	1.4:1	1.2:1	ND	2:1	1.4:1	1:1	ND

*ND* not described

In our study males were affected more as compared to females. We found positive correlation between gender and both nodal and extra nodal DLBCL. In both the groups males were affected approximately 1.5 times more than females and GIT was the commonest site of extra nodal DLBCL involvement. Both nodal and extra nodal DLBCL were more common in older age group, however no significant relationship was found between age and distribution of DLBCL. Females presented at comparatively lower age with DLBCL as compared to males. Research carried out by Joachim Yahalom et al. showed that extra nodal lymphomas comprised about one third of all NHL. They carried out research on various treatment options in extra nodal DLBCL showing that there must be different protocols for nodal and extra nodal DLBCL [19]. Shi Y et al. recruited 1085 Chinese patients of DLBCL 62% cases were nodal and 37% were extra nodal. The most common extra nodal site was stomach followed by intestine, nose and sinuses, testes, skin, thyroid, CNS, breast, bone, and salivary gland [21]. In our study the sites involved in extra nodal DLBCL in decreasing order of frequency were GIT, tonsil, spine, soft tissue, thyroid, breast and CNS. They found majority of extra nodal DLBCL presented with B symptoms and bulky disease in contrast to our study, in which we found more cases with Nodal DLBCL presented with B symptoms. However no statistically significant relationship was found between presence of B symptoms and nodal and extra nodal disease. Shen H et al. conducted a study on 141 patients with DLBCL. The primary extra nodal site involved was GIT followed by CNS, breast, adrenal glands, female genital system, thyroid and bone, similar to this study [22]. Dodan Yazilitas conducted research in Turkey. He enrolled 112 patients of Nodal NHL and 267 patients with extra nodal NHL. Similar to our studies three fourth of patients had stage 2 disease while 50% of extra nodal NHL had stage 1 disease at presentation.DLBCL was the most common histological subtype and GIT was the most common site of involvement (50%) followed by Head & Neck region (36%) [23].Another retrospective analysis was conducted in India over a period of 5 years It showed primary extra nodal NHL constituted 22% of all the cases and most common histological type was DLBCL with extra nodal subtype having a better overall prognosis than nodal variety [24]. As compared to nodal DLBCL extra nodal DLBCL patients were of older ages, and they presented at earlier stages (21%). A study was conducted in Pakistan to find out the prevalence and risk factors of DLBCL. They found that of all lymphomas,DLBCL comprises 86% of all the NHL emerging almost as an epidemic having nodal and extra nodal involvement and is rapidly fatal if not treated on time [25]. Another study conducted by Uzma et al. showed that out of 192 cases of NHL there were 113(68%) cases of DLBCL out of which 52% were found to be extra nodal which shows slightly higher percentage of extra nodal involvement as compared to our study, however similar to our study GIT was the most common site of extra nodal involvement [26]. A study conducted in Eden by Abdullah et al. showed frequency of DLBCL was 35% in their study. Most of the patients were nodal and B symptoms were present in 64% of them and approximately half of them had stage III & IV disease at presentation [28]. In conclusion DLBCL is the most common lymphoma in Pakistani population, especially targeting Northern areas with increased percentage of nodal DLBCL as compared to extra nodal DLBCL. Patients having extra nodal disease presented at earlier stages with fewer B symptoms in contrast to nodal DLBCL in which more patients had stage IV disease and B symptoms at presentation.

## Limitation

More studies on larger scale need to be conducted throughout the population of Pakistan for precise disease burden throughout the country.

This study provides food for thought for further studies that need to focus on finding etiological agents behind the alarming increase in lymphoma patients in Pakistan.

## Supplementary Information

Additional file 1: **Figure S1.** Age Statistics and Gender Distribution In DLBCL. **Figure S2.** Age Distribution in Nodal and Extra Nodal DLBCL. **Table S1.** Correlation between age and nodal and extra nodal DLBCL. **Table S2.** Correlation between male and female gender and nodal and extra nodal DLBCL **Figure S3.** Percentage of B Symptoms in nodal and extra nodal DLBCL. **Figure S4.** Percentage of stage 4 in nodal and extra nodal DLBCL. **Table S3.** Correlation between presence of B symptoms in nodal and extra nodal DLBCL. **Table S4.** Correlation between stage 4 disease and nodal and extra nodal DLBCL.

## Data Availability

The datasets generated and/or analyzed during the current study are not publicly available due to hospital policy on maintaining confidentiality and privacy of patient information but are available from the corresponding author on reasonable request.

## Abbreviations

NHL: Non-Hodgkin lymphoma
DLBCL: Diffuse Large B Cell lymphoma
GIT: Gastrointestinal tract
CNS: Central nervous system

## References

[1] Armitage JO, Gascoyne RD, Lunning MA, Cavalli F (2017). Non-Hodgkin lymphoma. Lancet.

[2] Shahid R, Gulzar R, Avesi L, Hassan S, Danish F, Mirza T (2016). Immunohistochemical profile of Hodgkin and non–Hodgkin lymphoma. J Coll Physicians Surg Pak.

[3] Ola K, Amany H, Yasser AS, Asmaa S, Mohammad A (2015). Clinicopathological profile and outcome of extranodal diffuse large B-cell NHL Egyptian National Cancer Institute Experience. Forum Clin Oncol.

[4] Menon MP, Pittaluga S, Jaffe ES (2012). The histological and biological spectrum of diffuse large B-cell lymphoma in the World Health Organization classification. Cancer J.

[5] Alyahya N, Adiga B, Alwadei A (2019). The clinico-pathological profile of non-Hodgkin’s lymphoma in Aseer region of Saudi Arabia. BMC Res Notes.

[6] Mertsoylu H, Muallaoglu S, Besen AA, Erdogdu S, Sezer A, Sedef AM (2014). Primary extranodal non-Hodgkins lymphoma: clinicopathological features, survival and treatment outcome in two cancer centers of southern Turkey. Asian Pac J Cancer Prev.

[7] Bangash MH, Hussain I, Zakaria M, Piracha MN (2014). Pattern Of extranodal involvement in non Hodgkins lymphoma. PAFMJ.

[8] Nawaz MZ, Bilal M, Mehmood MA, Asgher M (2015). Prevalence of Lymphoma Cancer in Punjab, Pakistan. Int J Appl Sci Biotechnol.

[9] Idrees R, Fatima S, Abdul-Ghafar J, Raheem A, Ahmad Z (2018). Cancer prevalence in Pakistan: meta-analysis of various published studies to determine variation in cancer figures resulting from marked population heterogeneity in different parts of the country. World J Surg Oncol.

[10] Zeb A, Rasool A, Nasreen S (2008). Cancer incidence in the districts of Dir (Northwest Frontier Province), Pakistan: a preliminary study. J Chin Med Assoc.

[11] Miranda-Filho A, Piñeros M, Znaor A, Marcos-Gragera R, Steliarova-Foucher E, Bray F (2019). Global patterns and trends in the incidence of non-Hodgkin lymphoma. Cancer Causes Control.

[12] Vannata B, Zucca E (2015). Primary extranodal B-cell lymphoma: current concepts and treatment strategies. Chin Clin Oncol.

[13] Sun Y, Qiao X, Jiang C, Liu S, Zhou Z (2020). Texture analysis improves the value of pretreatment ^18^F-FDG PET/CT in predicting interim response of primary gastrointestinal diffuse large B-cell lymphoma. Contrast Media Mol Imaging.

[14] Boussios S, Zerdes I, Vassou A, Bareta E, Seraj E, Papoudou-Bai A, Pavlidis N, Batistatou A, Pentheroudakis G (2018). Extranodal diffuse large B-cell lymphomas: a retrospective case series and review of the literature. Hematol Rep.

[15] Pai A, Kannan T, Balambika RG, Vasini V (2017). A study of clinical profile of primary extranodal lymphomas in a tertiary care institute in South India. Indian J Med Paediatr Oncol.

[16] Yin X, Xu A, Fan F, Huang Z, Cheng Q, Zhang L, Sun C, Hu Y (2019). Incidence and mortality trends and risk prediction nomogram for extranodal diffuse large B-cell lymphoma: an analysis of the surveillance, epidemiology, and end results database. Front Oncol.

[17] Smith A, Crouch S, Lax S, Li J, Painter D, Howell D (2015). Lymphoma incidence, survival and prevalence 2004–2014: sub-type analyses from UKs Haematological Malignancy Research Network. Br J Cancer.

[18] Chihara D, Oki Y, Fanale MA, Westin JR, Nastoupil LJ, Neelapu S, Fayad L, Fowler NH, Cheah CY (2019). Stage I non-Hodgkin lymphoma: difference in survival outcome by primary extranodal site of involvement. Br J Haematol.

[19] Yahlom J, Illlidge T, Specht L, Hoppe RT, Li YX, Tsang R (2015). Modern radiation therapy for extranodal lymphomas: field and dose guidelines from the International Lymphoma Radiation Oncology Group. Int J Radiat Oncol Biol Phys.

[20] Gogia A, Das CK, Kumar L, Sharma A, Tiwari A, Sharma MC, Mallick S (2018). Diffuse large B-cell lymphoma: an institutional analysis. South Asian J Cancer.

[21] Shi Y, Han Y, Yang J, Liu P, He X, Zhang C, Zhou S, Zhou L, Qin Y, Song Y, Liu Y, Wang S, Jin J, Gui L, Sun Y (2019). Clinical features and outcomes of diffuse large B-cell lymphoma based on nodal or extranodal primary sites of origin: analysis of 1085 WHO classified cases in a single institution in China. Chin J Cancer Res.

[22] Shen H, Wei Z, Zhou D, Zhang Y, Han X, Wang W, Zhang L, Yang C, Feng J (2018). Primary extra-nodal diffuse large B-cell lymphoma: a prognostic analysis of 141 patients. Oncol Lett.

[23] Yazilitas D, Ozdemir N, Hoazade C, Bozkaya Y, Yazici O, Sendur MA (2015). A retrospective comparison of early stage primary extranodal with nodal non–Hodgkin lymphoma patients: a single center experience. J BUON.

[24] Padhi S, Paul TR, Challa S, Prayaga AK, Rajappa S (2012). Primary extranodal non Hodgkin lymphoma: a 5 year retrospective analysis. Asian Pacific J Cancer Prev.

[25] Tariq A, Khurshid A, Mehmood Y (2015). Potential risk factors and prevalence trend of diffuse large beta cell lymphoma in Pakistani population. Int J Pharm Res Allied Sci.

[26] Bukhari U, Jamal S, Lateef F (2015). Non Hodgkin lymphoma—a study. Pak Oral Dental J.

[27] Waravita TS, Wijetunge TS, Ratnatunga NV (2015). Pattern of lymphoma subtypes in a cohort of Sri Lankan patients. Ceylon Med J.

[28] Abdullah AA, Bakhubaira SM, Al- Kahiry W, Moshara GH (2014). Pattern of diffuse large B cell lymphoma(DLBCL) in Aden, Yemen. Gulf J Oncol.

