# Supplementary material for: Clinicopathological spectrum of Diffuse Large B Cell lymphoma: a study targeting population yet unexplored in Pakistan
Source: BMC Res Notes. 2021 Sep 10;14:354. doi: 10.1186/s13104-021-05768-5 (PMC8434720; doi:10.1186/s13104-021-05768-5)
Supplement: Supplementary file 1 — Additional file 1: Figure S1. Age Statistics and Gender Distribution In DLBCL. Figure S2. Age Distribution in Nodal and Extra Nodal DLBCL. Table S1. Correlation between age and nodal and extra nodal DLBCL. Table S2. Correlation between male and female gender and nodal and extra nodal DLBCL Figure S3. Percentage of B Symptoms in nodal and extra nodal DLBCL. Figure S4. Percentage of stage 4 in nodal and extra nodal DLBCL. Table S3. Correlation between presence of B symptoms in nodal and extra nodal DLBCL. Table S4. Correlation between stage 4 disease and nodal and extra nodal DLBCL. [file 13104_2021_5768_MOESM1_ESM.docx]

**Supplementary File Containing Figures and Tables.**

Figure S1: Age Statistics and Gender Distribution In DLBCL


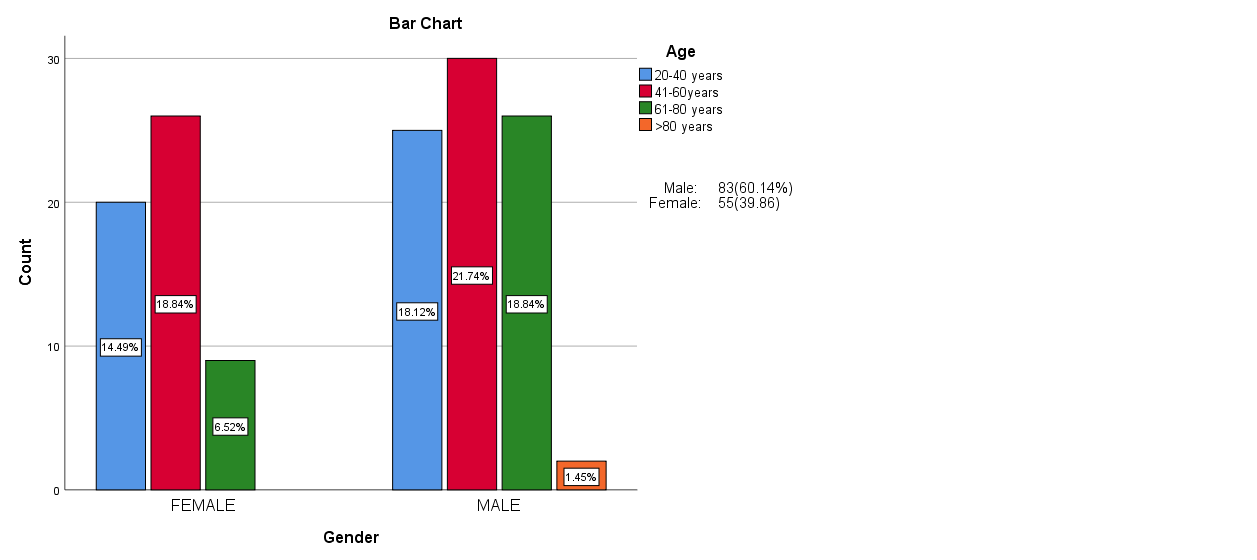


Figure S2: Age Distribution in Nodal and Extra Nodal DLBCL


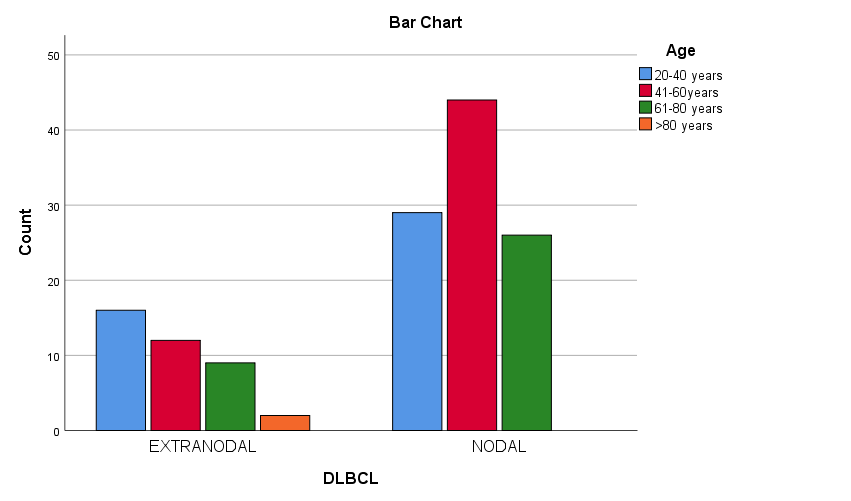


Table S1: Correlation between age and nodal and extra nodal DLBCL

**Chi-Square Tests**

|  | Value | Df | Asymptotic Significance (2-sided) |
| --- | --- | --- | --- |
| Pearson Chi-Square | 53.781^a^ | 37 | .037 |
| Likelihood Ratio | 65.121 | 37 | .003 |
| N of Valid Cases | 138 |  |  |
| a. 69 cells (90.8%) have expected count less than 5. The minimum expected count is .28. | | | |

Table S2: Correlation between male and female gender and nodal and extra nodal DLBCL

| **Chi-Square Tests** | | | | | |
| --- | --- | --- | --- | --- | --- |
|  | Value | df | Asymptotic Significance (2-sided) | Exact Sig. (2-sided) | Exact Sig. (1-sided) |
| Pearson Chi-Square | .044^a^ | 1 | .834 |  |  |
| Continuity Correction^b^ | .000 | 1 | .987 |  |  |
| Likelihood Ratio | .044 | 1 | .834 |  |  |
| Fisher's Exact Test |  |  |  | 1.000 | .496 |
| N of Valid Cases | 138 |  |  |  |  |
| a. 0 cells (.0%) have expected count less than 5. The minimum expected count is 15.54. | | | | | |
| b. Computed only for a 2x2 table | | | | | |

Figure S3: Percentage of B Symptoms in nodal and extra nodal DLBCL


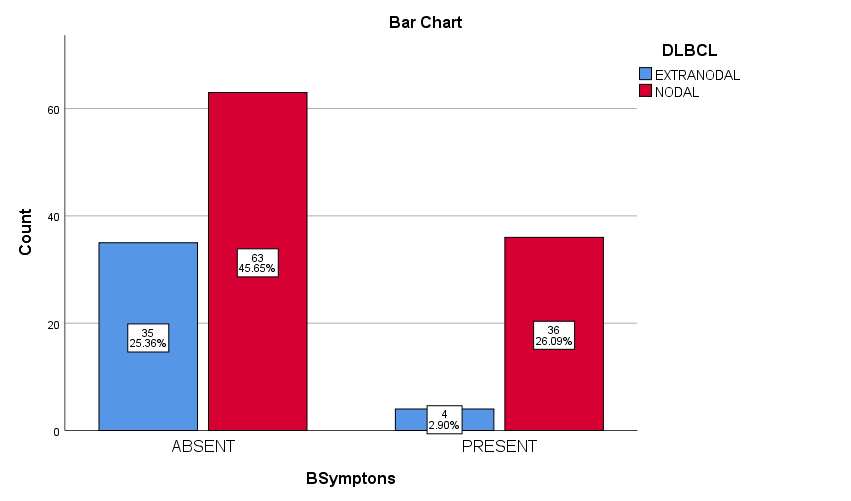


Figure S4: Percentage of stage 4 in nodal and extra nodal DLBCL


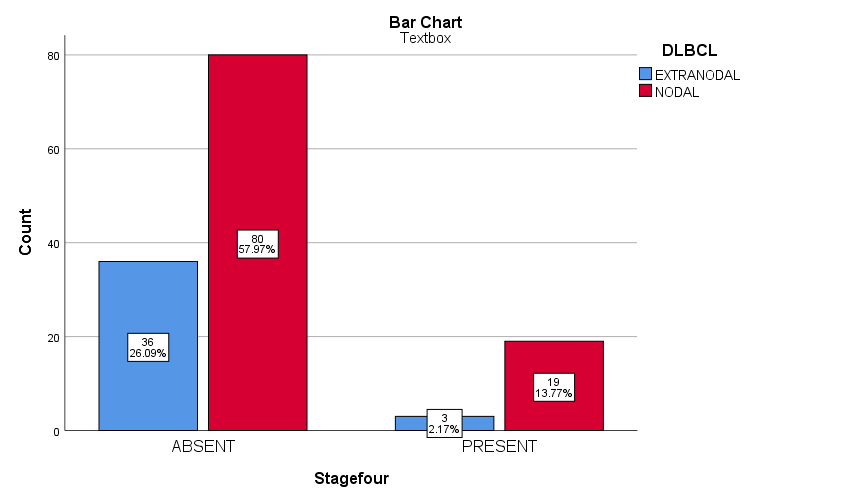


Table S3: Correlation between presence of B symptoms in nodal and extra nodal DLBCL

| **Chi-Square Tests** | | | | | |
| --- | --- | --- | --- | --- | --- |
|  | Value | Df | Asymptotic Significance (2-sided) | Exact Sig. (2-sided) | Exact Sig. (1-sided) |
| Pearson Chi-Square | 9.264^a^ | 1 | .002 |  |  |
| Continuity Correction^b^ | 8.039 | 1 | .005 |  |  |
| Likelihood Ratio | 10.580 | 1 | .001 |  |  |
| Fisher's Exact Test |  |  |  | .002 | .001 |
| N of Valid Cases | 138 |  |  |  |  |
| a. 0 cells (.0%) have expected count less than 5. The minimum expected count is 11.30. | | | | | |
| b. Computed only for a 2x2 table | | | | | |

Table S4: Correlation between stage 4 disease and nodal and extra nodal DLBCL.

| **Chi-Square Tests** | | | | | |
| --- | --- | --- | --- | --- | --- |
|  | Value | Df | Asymptotic Significance (2-sided) | Exact Sig. (2-sided) | Exact Sig. (1-sided) |
| Pearson Chi-Square | 2.761^a^ | 1 | .097 |  |  |
| Continuity Correction^b^ | 1.970 | 1 | .160 |  |  |
| Likelihood Ratio | 3.110 | 1 | .078 |  |  |
| Fisher's Exact Test |  |  |  | .124 | .075 |
| N of Valid Cases | 138 |  |  |  |  |
| a. 0 cells (.0%) have expected count less than 5. The minimum expected count is 6.22. | | | | | |
| b. Computed only for a 2x2 table | | | | | |
